# Innate immune markers that distinguish red deer (*Cervus elaphus*) selected for resistant or susceptible genotypes for Johne’s disease

**DOI:** 10.1186/1297-9716-44-5

**Published:** 2013-01-24

**Authors:** Brooke Dobson, Simon Liggett, Rory O’Brien, J Frank T Griffin

**Affiliations:** 1 Disease Research Laboratory, 720 Cumberland St, Dunedin 9016, New Zealand

## Abstract

While many factors contribute to resistance and susceptibility to infectious disease, a major component is the genotype of the host and the way in which it is expressed. Johne’s disease is a chronic inflammatory bowel disease affecting ruminants and is caused by infection with *Mycobacterium avium* subspecies *paratuberculosis* (MAP). We have previously identified red deer breeds (*Cervus elaphus*) that are resistant; have a low rate of MAP infection and do not progress to develop Johne’s disease. In contrast, susceptible breeds have a high rate of MAP infection as seen by seroconversion and progress to develop clinical Johne’s disease. The aim of this study was to determine if immunological differences exist between animals of resistant or susceptible breeds. Macrophage cultures were derived from the monocytes of deer genotypically defined as resistant or susceptible to the development of Johne’s disease. Following in vitro infection of the cells with MAP, the expression of candidate genes was assessed by quantitative PCR as well as infection rate and cell death rate. The results indicate that macrophages from susceptible animals show a significantly higher upregulation of inflammatory genes (iNOS, IL-1α, TNF-α and IL-23p19) than the macrophages from resistant animals. Cells from resistant animals had a higher rate of apoptosis at 24 hours post infection (hpi) compared to macrophages from susceptible animals. The excessive expression of inflammatory mRNA transcripts in susceptible animals could cause inefficient clearing of the mycobacterial organism and the establishment of disease. Controlled upregulation of inflammatory pathways coupled with programmed cell death in the macrophages of resistant animals may predispose the host to a protective immune response against this mycobacterial pathogen.

## Introduction

Understanding the protective immune response following mycobacterial infection is crucial to the design of preventive treatments, improved diagnostics and vaccines. The majority of immunological data on this topic is derived from studies of inbred strains of laboratory mice. However, experiments designed to characterise this immune response should consider the genotypic heterogeneity of outbred populations and how this could impact on responses to mycobacterial pathogens.

Johne’s disease is an inflammatory bowel disease of ruminants caused by infection with *Mycobacterium avium* subspecies *paratuberculosis* (MAP). Red deer (*Cervus elaphus*) are natural hosts of MAP and are prone to develop Johne’s disease at a young age [1]. Johne’s disease conforms to the classical “iceberg effect” seen with infectious diseases, where a large proportion of animals may be infected with MAP but only a small proportion of animals develop pathology or clinical disease [2], demonstrating that the microorganism itself is necessary but not sufficient for the development of Johne’s disease. Individuals within a host population may display a spectrum of infection outcomes ranging from resistant to susceptible under certain environmental conditions. A wide spectrum of resistance and susceptibility to mycobacterial disease has been observed within human populations and in-bred mouse strains [3]. A previous study [4] identified breed-lines of red deer that exhibited polarised susceptibility or resistance to tuberculosis caused by *M. bovis*. Heritability of these phenotypes in progeny bred from resistant or susceptible sires was high (0.48 +/− 0.10). Heritability for tuberculosis in Irish and British dairy cows has been reported in the range of 0.04 to 0.27 [5,6].

A number of scientific studies infer that there are breed differences in the susceptibility to Johne’s disease of cattle [7], sheep [8,9] and deer [10] suggesting a genetic component to susceptibility. Our laboratory has identified breed-lines of deer that express a resistant or susceptible phenotype for Johne’s disease on a large stud farm that had eight distinct breeds of deer that were derived from stags and hinds imported from disparate herds throughout Europe. The heritability of Johne’s disease resistance or susceptibility in this stud herd was estimated to be 0.30 +/− 0.06 (J.F.T Griffin, Disease Research Laboratory, unpublished data) whereas heritability in crossbred deer herds has been estimated to be 0.2 [11]. An estimated heritability for susceptibility to MAP infection in a herd of Dutch dairy cattle was lower at 0.06 [7] while heritability for the production of antibody to MAP was estimated at 0.15 for Irish dairy cows [12] and 0.10 for Danish dairy cows [13]. Moreover, findings from Johne’s disease research in sheep suggest that a resistant phenotype exists where the animal prevents the establishment of MAP infection [14] or the infected animal is able to cure itself over a period of time [15].

The heritability of resistance and susceptibility in different deer genotypes was confirmed by an experimental MAP challenge study using the progeny of sires selected from breeds that display either a resistant or susceptible phenotype [10]. Striking differences were observed between the two groups of deer where seven of nine progeny from a resistant sire displayed a resistant phenotype in contrast to eight of nine progeny from a susceptible sire which expressed a susceptible phenotype. Disease status was determined by the severity of histopathology, detection of MAP in infected lymph nodes and immunodiagnostic markers such as serum IFN-γ and antibody (Paralisa™) levels [10].

A focus of many studies of mycobacterial pathogens is the macrophage as this cell plays a central role in both the innate and adaptive immune response to mycobacteria. Several studies have investigated the immune response of bovine macrophages to MAP infection and contrasted the response of macrophages from animals which are not infected with those that are infected or diseased [16-18]. Within an infected group, there will typically be those that will eventually develop Johne’s disease (susceptible) and those that contain or clear the infection (resistant), similar to what is seen from experimental infection trials [10,15]. We hypothesised that uninfected red deer that have either a resistant or susceptible genotype may be distinguished by the differential expression of candidate genes in macrophages following infection with MAP in vitro.

A group of purebred animals where breed integrity had been preserved, and a group of crossbred animals of more heterogeneous genotypes, were sampled to determine if differences existed between resistant and susceptible animals in terms of the immune response to in vitro MAP infection. Quantitative PCR (Q-PCR) was used to analyse the expression of a panel of candidate genes associated with the innate immune response to mycobacteria in macrophages.

Inducible nitric oxide synthase (iNOS) was chosen as a target gene as it has been reported to play a role in the bovine monocyte-derived macrophage (MDM) response to MAP [18]. IL-α, TNF-α and IL-6 are key pro-inflammatory cytokines associated with macrophage function and have been described as important molecules in the innate response to mycobacterial pathogens [19,20]. IL-10 is an anti-inflammatory, regulatory cytokine that is produced by macrophages to reduce excessive inflammation and its expression is thought to play a role in both protecting the host from MAP-related pathology and the subversion of the immune response by the microorganism [17]. The inflammatory cytokine IL-23 is integral to the function of Th17 cells which are involved in the pathology of human inflammatory bowel disease, particularly Crohn’s disease [21] and have also been implicated in the pathology of Johne’s disease [22]. Finally, IL-12, which is a key molecule involved in the Th1 pathway considered to provide protection against mycobacterial disease [23] was included in the study. Cell death and infection rates were also assessed in cultured macrophages in response to MAP infection.

The identification of animals prone to developing Johne’s disease or other mycobacterial diseases may enable the tailoring of treatment and management programs for these conditions. The ultimate goal of this study would be to identify laboratory markers which could be used diagnostically to select sires or dams in foundation breeding programs to develop resistant herds. Furthermore, elucidation of the protective immune response to mycobacterial pathogens seen in resistant animals would also be informative for future vaccine development by providing correlates of protection and markers to test vaccine efficacy.

## Materials and methods

### Sample collection

Bulk blood samples (300 mL) were collected from all animals by jugular venepuncture by trained, experienced technicians or veterinarians into citrate phosphate dextrose-containing blood bags (Pharmaco, New Zealand). All experimental manipulations on animals were carried out under ethical approval from the Invermay AgResearch Animal Ethics Committee (AE#12102).

**Figure 1:**
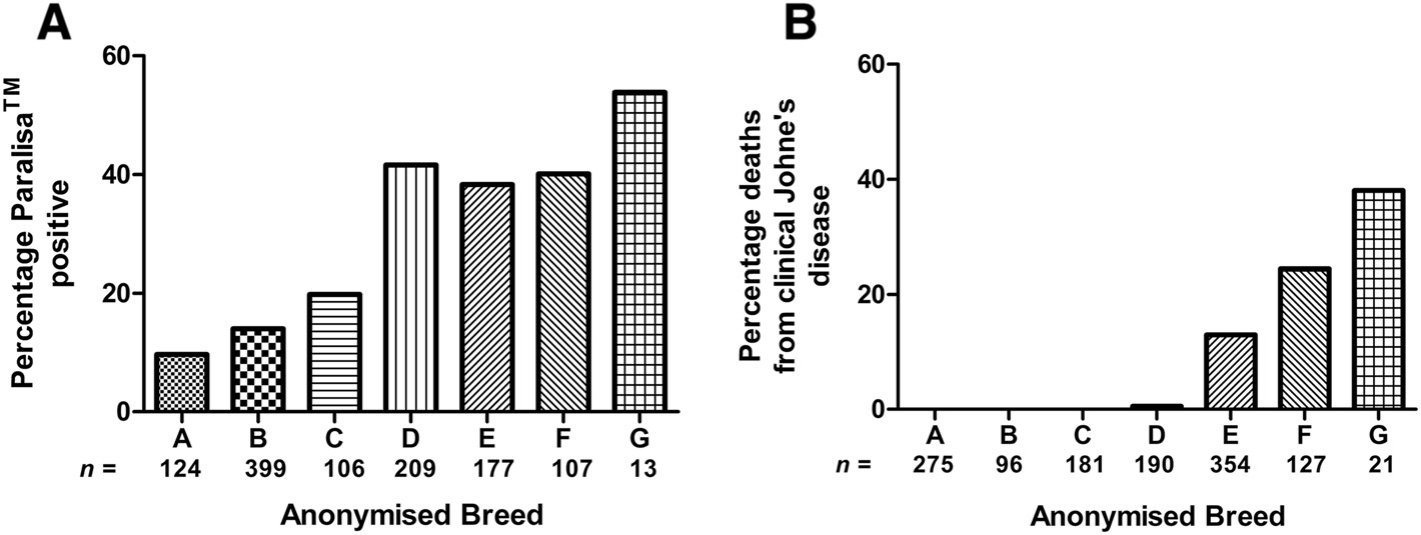
**Resistance and susceptibility to Johne’s disease in a red deer stud herd.** (**A**) Diagnosis of MAP infection by Paralisa™ in animals of distinct pure breeds (anonymised to A – G, *n* = 1135 animals total). Differences in reactivity rate are statistically significant (*p* < 0.01) between breeds with the exception of differences between Breeds A, C and G. (**B**) Deaths from clinical Johne’s disease in the different pure breeds (A – G) recorded over a 6 year period prior to serological testing (*n* = 1244 animals total). Differences in death rate are statistically significant (*p* < 0.01) between breeds A – C and breeds E – G. A high level of exposure of all animals confirms that the resistant breeds, with low incidence of MAP infection and no deaths from Johne’s disease (breeds A – C), have a resistant phenotype and do not include unexposed indeterminate animals.

Two groups of animals were sampled for these experiments. The first group of animals comprised 20 purebred, one year old animals from a deer stud farm. The herd had experienced high levels of exposure to MAP from 1999 onwards. When serological monitoring began in 2004, there was a high level of infection (> 20%) with significant numbers of animals dying from clinical Johne’s disease between 2000 and 2004. The stud herd contained seven distinct deer breeds, some of which historically displayed extremes of resistance or susceptibility to Johne’s disease after chronic environmental MAP exposure. This is demonstrated in Figure 1 where serological testing, measuring MAP-specific antibody levels (Paralisa™ test [24]), was undertaken on 1135 animals of different breeds and deaths from clinical Johne’s disease recorded over a 6 year period before serological testing commenced (Figure 1). An extensive database of breed value parameters, pedigree and infection/disease status, as measured by Paralisa™ and confirmed by histopathology following necropsy, was available for all the animals born on this property from 2000 onwards. The whole herd genotype database was analysed to generate Johne’s Breed Values (JBV) which resulted in a probability score that an individual animal would resist infection or develop clinical Johne’s disease and become Paralisa^TM^ positive following exposure to MAP. In order to generate JBV, a Johne’s disease presence/absence trait was created for every animal on the property. Animals that were culled or died from clinical Johne’s disease and animals with a positive Paralisa result were given a value of 1 and the remaining animals with negative Paralisa results were given a value of 0. Animal-model restricted maximum likelihood (REML) analyses were then used [25], to estimate heritabilities and genetic correlations, with a repeated-record model. A positive JBV (0.1 to 0.5) implies that an animal is susceptible to infection whereas a negative JBV (−0.1 to −0.5) is indicative of resistance to Johne’s disease and the likelihood that they will not seroconvert following exposure to MAP. The JBV was calculated using existing Paralisa™ and clinical Johne’s disease incidence of progeny born on the property since 2000. The experimental group comprised 20 yearling animals including 10 animals of susceptible genotype (dam or sire JBV of greater than 0.3) and 10 animals of resistant genotype (dam or sire JBV of less than−0.3). In this purebred group of animals, there were ten females and ten males which were all Paralisa™ negative and considered uninfected at the time of testing. Peripheral blood samples from these animals were used for gene expression experiments.

The second group of animals comprised 13 animals and were selected to represent cross-bred animals with a predicted resistant or susceptible genotype based on historical breed and sire information. They were the progeny of an artificial insemination programme using semen from three stags. The animals of resistant genotype were all the progeny of one sire of a resistant breed which has historically hadvery low incidence of clinical Johne’s disease or serological reactivity (breed B in Figure 1). Animals considered to have susceptible genotypes were the progeny of either of two sires, both of mixed breeds associated with susceptibility as observed from high clinical Johne’s disease incidence and serological reactivity rates (breeds D – G in Figure 1). As the dams were outbred females of indeterminate genotype and mixed breed, the genotypes of the progeny were specified by linkage to the paternal genetics. The herd of origin had a history of very low levels of MAP infection so the risk of any of the experimental animals being infected was negligible. These animals were sampled at 5–6 months of age and at the time of sampling were all Paralisa^TM^ negative. Samples from these animals were used for gene expression experiments and cell death detection experiments.

### Monocyte-derived macrophage culture

Whole blood was mixed 1:1 with cold citrated RPMI (3.83 g tri-sodium citrate per litre) (Gibco, USA). The blood/RPMI mixture was layered over Histopaque 1083 (Sigma-Aldrich, USA) and centrifuged for 20 min at 600 *g*. Mononuclear cells at the interface were removed by aspiration into centrifuge tubes (Becton Dickinson, USA) containing citrated RPMI and centrifuged for 15 min at 500 *g.* A wash step followed, which involved resuspending the cells in citrated RPMI, centrifugation at 500 *g* for 15 min and then resuspending in RPMI (Invitrogen, USA) supplemented with 4 mM L-Glutamine. Cells were counted in a Countess^®^ Automated Cell Counter (Invitrogen, USA) and the concentration adjusted to 4 × 10^6^ live cells per mL. Cell suspensions were aliquoted into slide flasks (Nunc, Germany) for cell death detection experiments and 25 cm^2^ vented cell culture flasks (Becton Dickinson, USA) for gene expression experiments.

**Figure 2:**
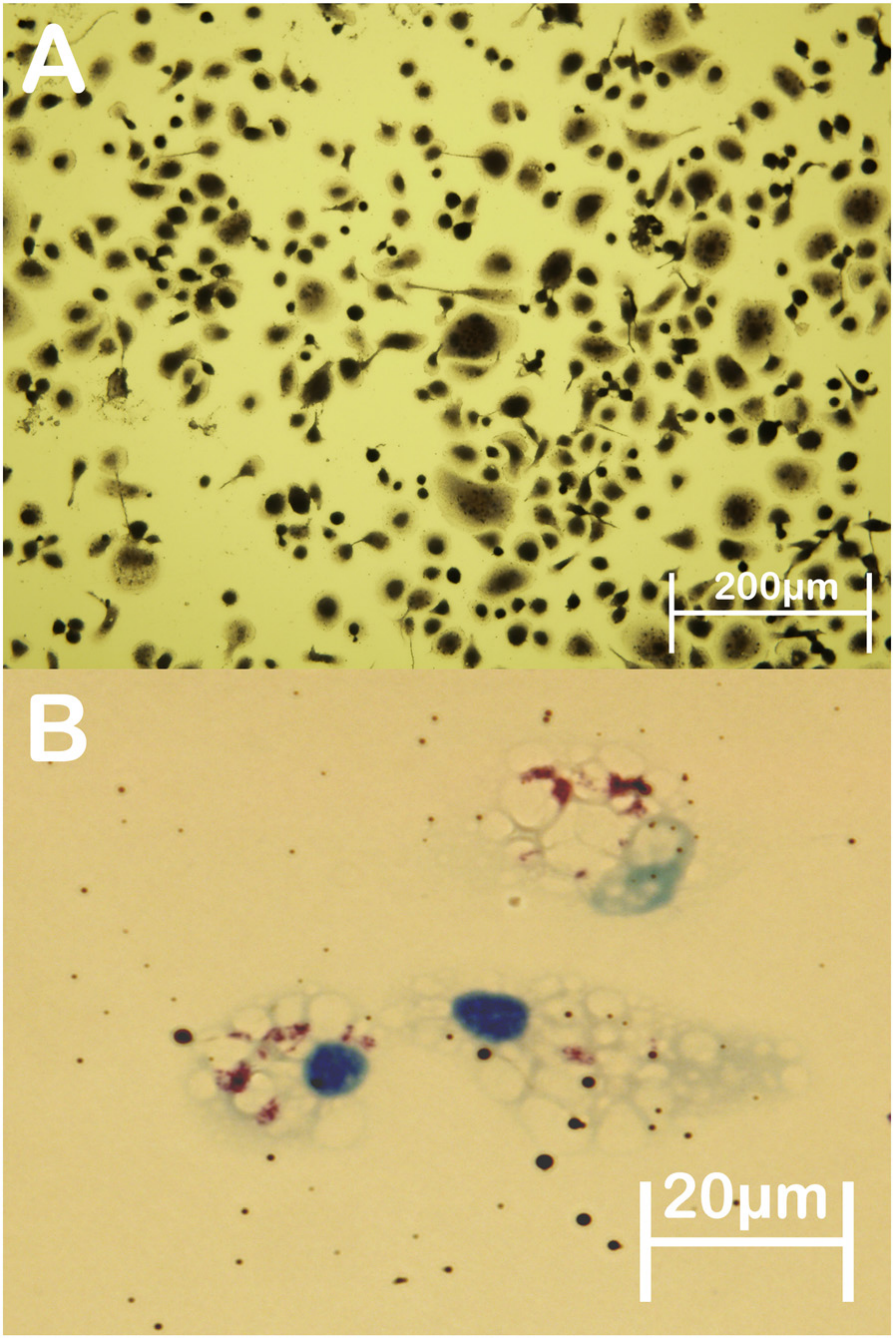
**Characteristics of mature deer monocyte-derived macrophages. **(**A**) Monocyte-derived macrophages stained positive (black) for α-naphthyl esterase at day 7 of culture (20X magnification). This enzyme is detected primarily in monocytes and macrophages and is virtually absent from granulocytes and other leukocytes, confirming that these adherent cells derived from deer blood are of a monocytic lineage. (**B**) Ziehl-Nielsen stain of deer MDM (blue nucleus) which has phagocytosed MAP bacilli; red, acid-fast microorganisms (100X magnification).

The cell culture vessels were incubated at 39°C in 5%CO_2_ for 2 h to select for monocytic cells that adhered to the plastic surface. Media was removed and the cells were washed once with PBS followed by the addition of RPMI containing 10% deer serum obtained from healthy mixed age animals. The culture vessels were incubated for 24 h at 39°C/5%CO_2_ and then were washed thoroughly with PBS before addition of 10% deer serum RPMI culture media. The macrophage cultures were matured for 7 days by which time the cells had taken on the characteristic morphology of mature macrophages in culture. Confirmation of macrophage phenotype was undertaken by α-naphthyl acetate esterase staining (Figure 2A), CD14 cell surface expression by flow cytometry using the monoclonal antibody CAM36A (Veterinary Medical Research & Development Inc., Washington State University, USA) as previously described [26]. The recovery of CD14+ cells from different animals ranged from 77 to 90%. Functional assays including phagocytosis detection (Figure 2B) and microbicidal activity following *E. coli* infection (data not shown) also confirmed these cells had the functional capacity of macrophages. Macrophages were counted on the Olympus IX-71 inverted microscope (Olympus, Japan) prior to infection experiments.

### Infection of MDM

The bacterial strain used to infect the macrophage cultures was *Mycobacterium avium* subspecies *paratuberculosis* K10. This strain was chosen because it is a virulent, low passage clinical bovine strain, typical of the strains identified as causing Johne’s disease in red deer [27]. The MAP culture was incubated in Middlebrook 7H9 medium (Fort Richard Laboratories, NZ) supplemented with OADC and mycobactin J (Allied Monitor, USA) and slowly agitated on a stirrer at 37°C for 10 days before enumeration by light microscopy. Maintaining the MAP culture on a stirrer minimized clumping and facilitated enumeration (cell numbers were confirmed to be accurate by plate culture). At day 7 of deer MDM culture, the MDM were washed twice with warm PBS and 10% deer serum RPMI, without gentamicin, was added to the cells. The cells were subsequently infected with MAP at a multiplicity of infection (MOI) of 10:1. The tissue culture vessels were incubated for 2 h at 39°C/5%CO_2_ before washing twice with warm PBS and replacing the gentamicin-free media with 10% deer serum RPMI with gentamicin. The tissue culture vessels were then incubated for a further 22 h (gene expression and cell death detection experiments, 24 h time point) or 46 h (cell death detection experiments, 48 h time point). For gene expression experiments, media was removed and 500 μL ISOLATE RNA Lysis Buffer (Bioline, USA) was added to the culture vessels. For cell death detection experiments, media was removed and stored at −20°C before preparing the slides for staining.

### RNA extraction and cDNA synthesis

The SuperScript^®^ VILO™ cDNA Synthesis Kit (Invitrogen, USA) was used according to the manufacturer’s instructions to synthesize first strand cDNA from the mRNA in the total RNA sample. Total RNA amounts reverse transcribed were normalised among individual animals. The resulting cDNA was diluted 1 in 4 with water and stored at −20°C until use in Q-PCR experiments.

### Quantitative PCR

In this work, efforts have been made to adhere to the Minimum Information for Publication of Quantitative Real-Time PCR Experiments (MIQE) guidelines as advocated by Bustin [28,29] in the reporting of Q-PCR methodology and results. Using the NormFinder [30] and geNorm [31] algorithms, β-2 microglobulin (B2M) and peptidyl-prolyl cis-trans isomerase B (PPIB) were selected as appropriate reference genes. Q-PCR primers had previously been developed for some of the target genes (IL-10, IL-1α, TNF-α, IL-23p19 and B2M) [22]. For those genes that had not been investigated (iNOS and PPIB), primers were designed as follows. Cervine sequence information was obtained from the AgResearch Cervine Genome Database using the coding sequence information of the bovine homologue and a BLAST-like tool. Bovine splice sites were identified from the UCSC Genome Browser [32] and mapped to the corresponding location in the cervine sequence. Primer Express version 2.0 software (Applied Biosystems, USA) was used to design primers that would span the marked splice sites and so prevent possible amplification of genomic copies of the target gene. Primers were synthesised at a commercial facility (Sigma Aldrich, USA). Information for all primers for reference and target genes is presented in Table 1. All primers for reference and target genes were confirmed to amplify a single product of the expected size according to gel electrophoresis and dissociation curves (data not shown).

**Table 1:** ***Cervus elaphus *primer sequences**

**Target**	**Primer sequence (5' to 3')**	**Amplicon size (bp)**	**Accession number**
Β2M forward	GGCTGCTGTCGCTGTCT	75	DQ482731.1
Β2M reverse	TCTGGTGGGTGTCTTGAGTACA		
PPIB forward	TGGCTACAAAGACAGCAAATTCC	148	NM_174152.2*
PPIB reverse	CCAGGCCCATAATGTTTAAGCT		
iNOS forward	GAAGAGGCTGAGAAGCAGAGGTT	98	NM_001076799.1*
iNOS reverse	TCCAGCACCTCCAGGAATGT		
IL-1α forward	ATCCACGAGGAATGCATCCT	147	EU860100.1
IL-1α reverse	AGAATCCTCTTCTGATACATAAGCAACA		
TNF-α forward	AGGGAAGAGCAATCCCCAACT	123	U14683.1
TNF-α reverse	CTGAGCGTTGATGTTGGCTACA		
IL-23p19 forward	GATGTCCCCCGTATCCAGTGT	114	EU860097.1
IL-23p19 reverse	CAGCAGCTTCTCGTAAAAAACCA		
IL-12p35 forward	GCCTCAACTACTCCCAAAACCT	83	U57751.1
IL-12p35 reverse	GCAGGAGTAAAATTCTAGGGTTTGTC		
IL-10 forward	CGGTGGAGCAGGTGAAGAG	71	U11767.1
IL-10 reverse	AAACTCACTCATGGCTTTGTAGACA		

* Accession number for corresponding bovine gene which was used to BLAST search the AgResearch Cervine Sequence Database to obtain cervine sequence from which to design primers.

Q-PCR was carried out in a 96-well format in 15 μL reaction volumes per well. PCRs were performed in duplicate using ABsolute™ QPCR SYBR^®^ Green mix (ABgene, UK), with amplification primers at 70nM and 2 μL diluted cDNA. The plates were analysed by the 7500 Fast Real-Time PCR System (Applied Biosystems, USA). Thermal cycling conditions consisted of enzyme activation at 95°C for 15 min, followed by 40 cycles of denaturation at 95°C for 15 s and annealing and extension at 60°C for 60 s. Post-PCR dissociation melting curves were determined for every reaction to confirm specificity and melting temperature of the amplification products (data not shown). Relative gene expression was calculated using the ∆ ∆ Ct method [33,34]. For each sample, the difference in Ct values between the target gene and reference genes (the average of B2M and PPIB) was calculated (the ∆ Ct). Relative gene expression after infection (or fold change) was then calculated by subtracting the uninfected control sample ∆ Ct from the infected sample ∆ Ct to yield the ∆ ∆ Ct. PCR efficiencies for every Q-PCR experiment were calculated by linear regression of individual amplification plots using LinRegPCR software [35]. The negative value of the ∆ ∆ Ct was subsequently used as an exponent of the calculated PCR efficiency, resulting in fold changes of expression in the treated sample relative to the untreated control sample.

### Cell death detection

DNA fragmentation as a measure of apoptosis was assessed by the in situ terminal deoxynucleotidyltransferase-mediated dUTP-biotin nick end labelling (TUNEL) technique using an in situ *c*ell death detection kit (TMR red; Roche Applied Science, Switzerland). Following 24 or 48 h of MAP infection, culture media was removed and the cells were fixed with 4% paraformaldehyde in PBS (pH 7.4) before TUNEL staining was performed according to the manufacturer’s instructions. MAP organisms were stained using the TB Fluorescent Stain Kit M (Becton Dickinson, USA) according to the manufacturer’s instructions followed by counterstaining of cell nuclei using Hoechst 33342 at 2 μg/mL (Invitrogen, USA). The slides were rinsed in water before ProLong^®^ Gold Antifade Reagent (Invitrogen, Life Technologies, USA) and coverslips were placed on the slides. At least 75 cells were counted for each animal and treatment on an Olympus BX-510 upright microscope (Olympus, Japan). The researcher was blinded to the genotypes of the animals from which the samples were obtained as well as the treatment of the cells. The number of cells infected with MAP and TUNEL positive was counted and recorded. Infected cells were further recorded as containing less than 10 MAP per cell, 10 – 20 MAP per cell or greater than 20 MAP per cell. The amount of lactate dehydrogenase (LDH) released into the supernatant of cell cultures 24 or 48 h after MAP infection was used to determine death from necrosis and was measured using the cytotoxicity detection kit (LDH; Roche Applied Science, Switzerland) according to the manufacturer’s instructions. The absorbance of the samples was measured at 490 nm, with a reference wavelength of 650 nm, using an ELISA plate reader (Bio-Rad Model 3550 Microplate Reader, Japan). Uninfected macrophages treated for 4 to 6 h with 200 ng/mL staurosporine (Sigma-Aldrich, USA) were included as a positive control for cell death.

### Statistical analysis

Significant differences between proportions (as in Figure 1A) were calculated using the z-test for proportions. To determine statistically significant differences in relative gene expression between the resistant group and the susceptible group, the Mann–Whitney test was performed on fold change values. Results for all other experiments with two or more groups or treatments were compared by the Kruskal-Wallis test and significant differences were calculated by Dunn’s post test. Significance is expressed as *p* < 0.05, *p* < 0.01 or *p* < 0.001 as described.

## Results

**Figure 3:**
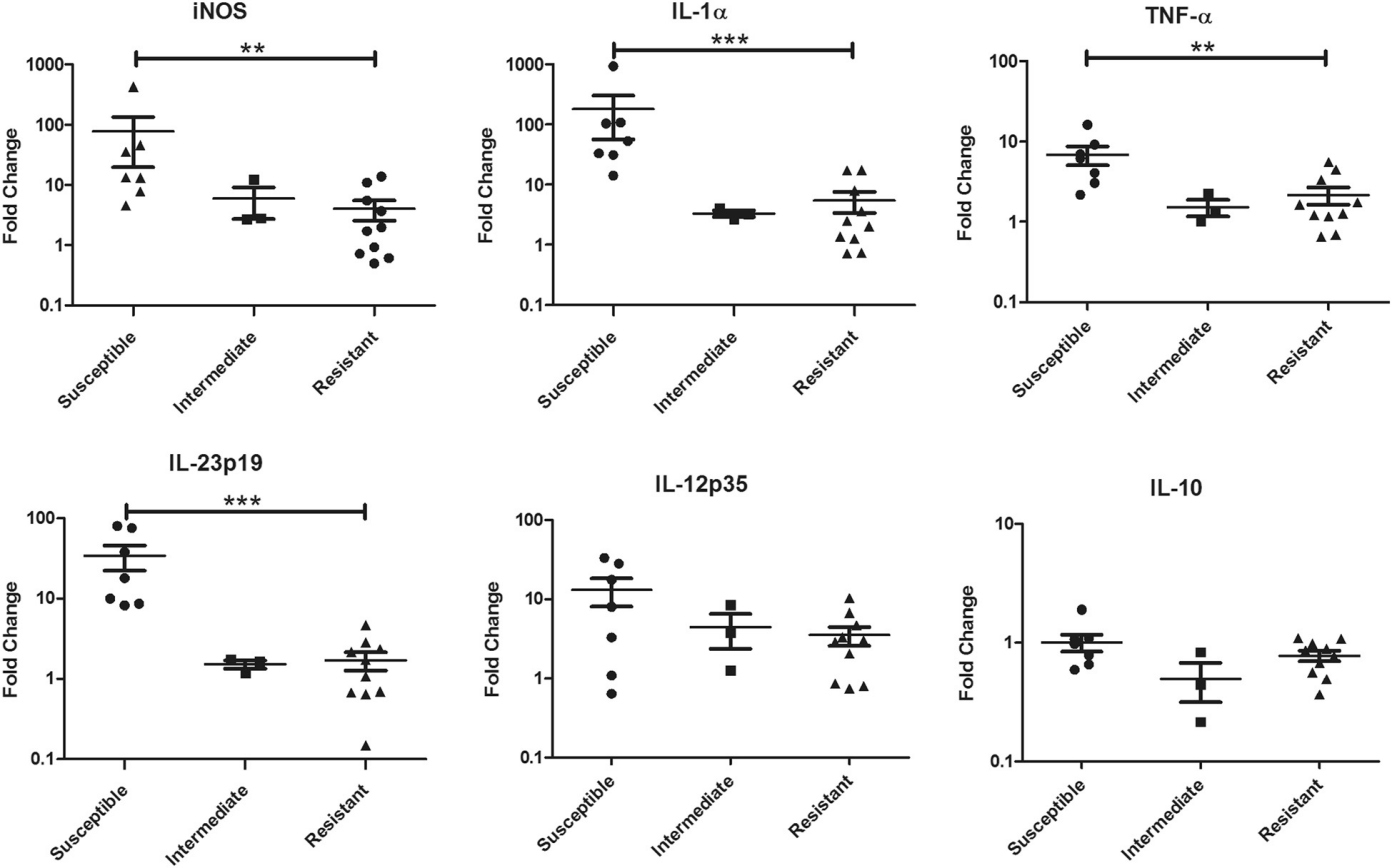
**Relative mRNA transcript expression of MDM from purebred animals of a resistant or susceptible genotype. **MDM were infected with MAP for 24 h before expression of the genes iNOS, IL-1α, TNF-α, IL-23p19, IL-12p35 and IL-10 was measured by Q-PCR. Fold changes (mean ± SEM) are defined as expression upon infection compared to the uninfected control, *n* = 20 (10 resistant, 3 intermediate and 7 susceptible). Three animals of susceptible genotype were removed from the susceptible group into an “intermediate” category on observing that their macrophage gene expression profiles resembled a resistant-type animal for the iNOS, IL-1α, TNF-α and IL-23p19 molecules. Statistical significance has been calculated using the Mann–Whitney test where ** denotes *p* < 0.01 and *** denotes *p* < 0.001.

When MAP was added to the macrophages isolated from purebred red deer, the expression of most of the genes was altered and relative gene expression differed between resistant and susceptible animals (Figure 3). Macrophages from susceptible animals upregulated the expression of the inflammatory molecules TNF-α (*p* < 0.01), iNOS (*p* < 0.01), IL-1α (*p* < 0.001) and IL-23p19 (*p* < 0.001) significantly more than did macrophages from resistant animals (Figure 3). Within the original group of ten susceptible animals, there were three animals which clustered together and displayed a resistant-like gene expression profile in the iNOS, IL-1α and IL-23p19 plots. These animals are the representative progeny of a single sire with a JBV close to neutral (0.04) and have been placed in a separate “intermediate” category in Figure 3. The low sire JBV of these animals indicates no paternal bias towards a resistant or susceptible genotype thus the genotype of these animals which resulted in their original inclusion in the susceptible group has been contributed by their respective dams (JBV > 0.3). The differences in gene expression between the resistant and susceptible groups remain significant (*p* > 0.01) for the iNOS, IL-1α and IL-23p19 molecules even when these three outlier animals are included in the susceptible group (data not shown).

While there was a trend for greater upregulation of IL-12p35 in macrophages from animals with a susceptible genotype compared with macrophages from resistant animals, this was not statistically significant. There was also no difference in the relative expression levels for IL-10 in response to MAP infection between the groups (Figure 3). Indeed, the mean fold change of IL-10 after 24 h of MAP infection was close to 1 in both the resistant- and susceptible-type macrophages (0.78 and 0.86 respectively), indicating that there was no change in IL-10 gene expression levels, compared to the untreated control at this time point.

**Figure 4:**
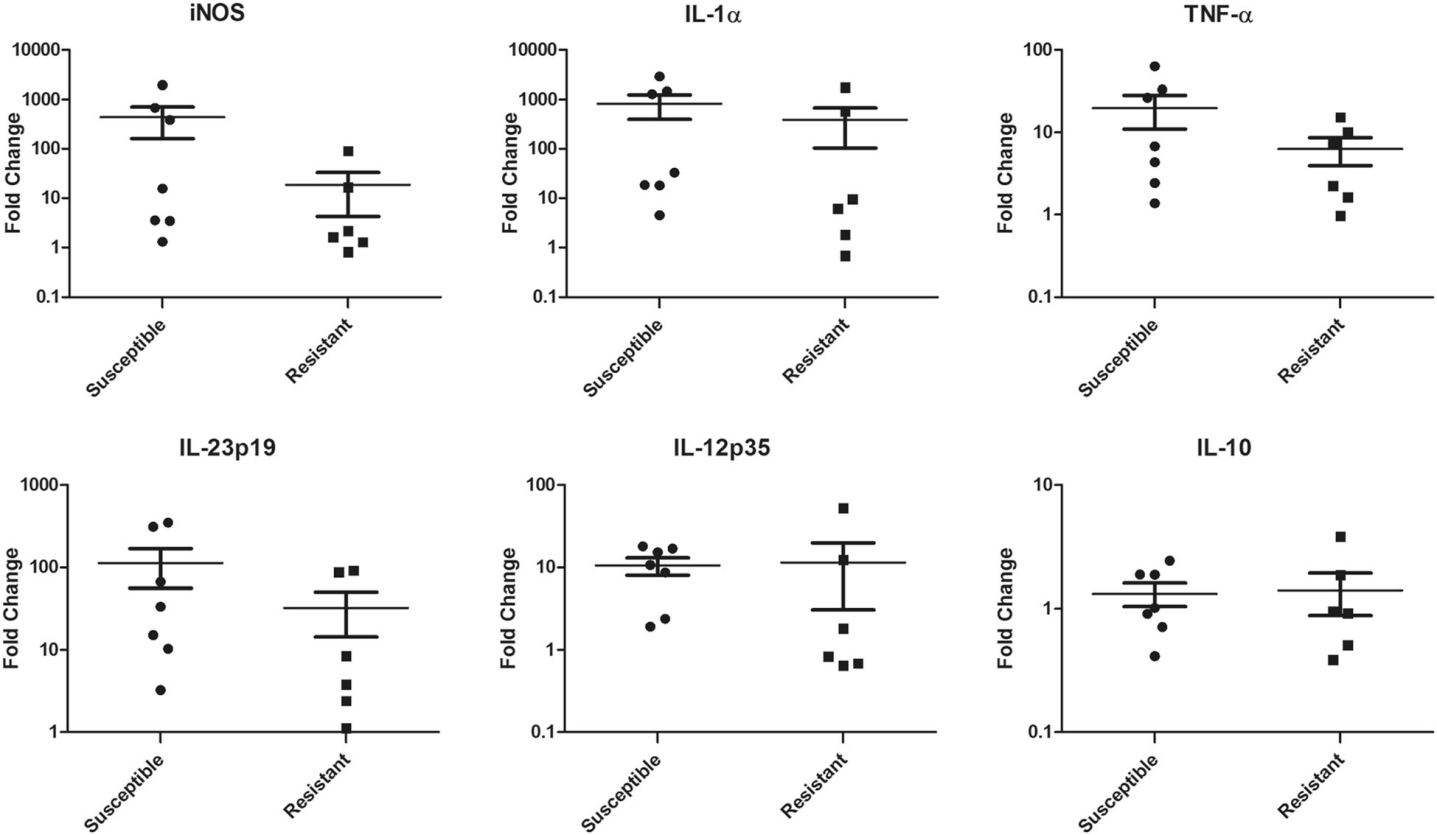
**Relative mRNA transcript expression of MDM from crossbred animals of a resistant or susceptible genotype. **MDM were infected with MAP for 24 h before expression of the genes iNOS, IL-1α, TNF-α, IL-23p19, IL-12p35 and IL-10 was measured by Q-PCR. Fold changes (mean ± SEM) are defined as levels of expression after infection compared to the uninfected control, *n* = 13 (6 resistant and 7 susceptible). No statistically significant differences were observed between the resistant and susceptible crossbred groups.

The patterns of gene expression in macrophages from crossbred animals infected with MAP in vitro were similar to that of macrophages from purebred animals but did not reach statistical significance (Figure 4). The mean upregulation of the inflammatory gene targets (iNOS, IL-1α, TNF-α and IL-23p19) in the macrophages from animals with susceptible genotypes was higher than that of cells from the animals of resistant genotypes. The cytokines IL-12p35 and IL-10 again did not differentiate the groups. The range of fold change values seen in this crossbred group of animals was also similar to that of the purebred animals although there was a larger scatter of values evident for these animals than was seen with the purebred animals.

**Figure 5:**
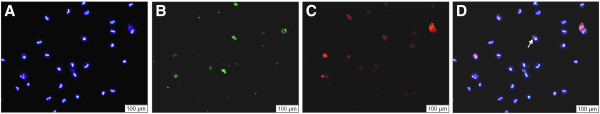
**Fluorescent microscopy images. **(**A**) Hoechst 33342 Stain (Nucleus), (**B**) TB Auramine M Stain (acid-fast MAP), (**C**) TUNEL TMR Red Stain (Apoptosis), (**D**) Overlay of the images, arrow pointing to macrophage containing an acid-fast bacilli (20X magnification).

**Figure 6:**
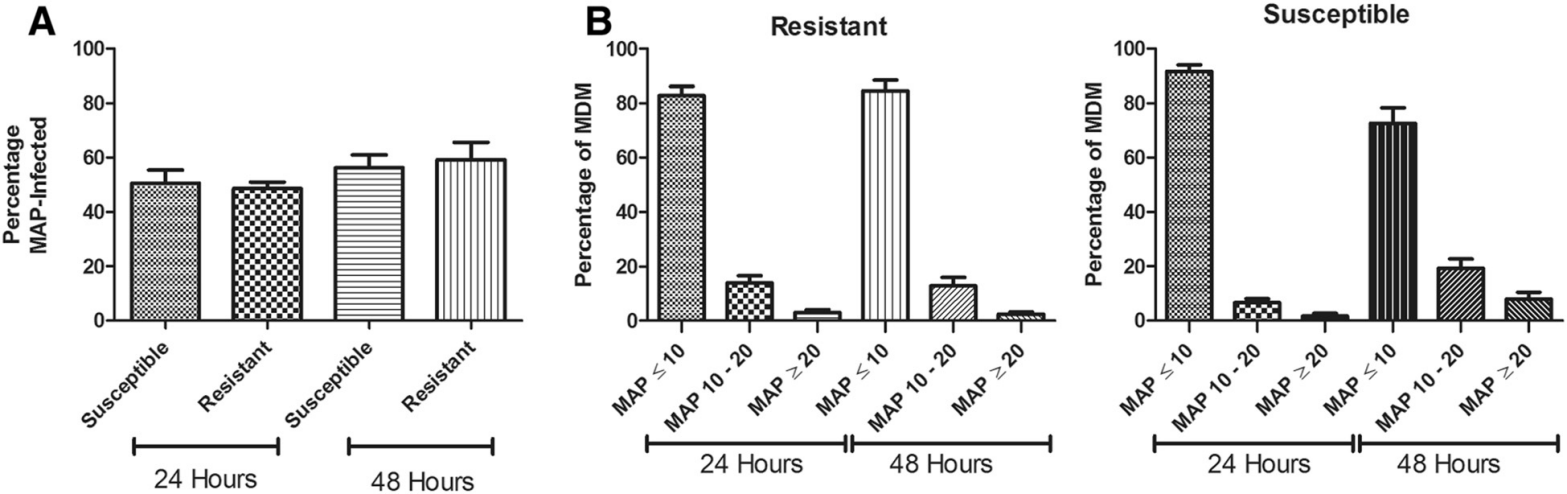
**Infection dynamics of MDM from resistant or susceptible deer, infected with MAP in vitro. **(**A**) Infection rate of macrophages from crossbred red deer of a resistant or susceptible genotype. MDM were isolated from crossbred red deer and cultured for 7 days on slide flasks. At day 7 of MDM culture, the cells were infected with MAP (MOI of 10:1) for 24 or 48 h and the resulting MAP infection rate was determined by auramine staining. The data are expressed as percentages of MAP-infected cells (mean ± SEM) and at least 75 cells per slide were counted, *n* = 13 (6 resistant and 7 susceptible). (**B**) Numbers of MAP bacilli per infected macrophage from crossbred red deer of a resistant or susceptible genotype. The data are expressed as percentages of MAP-infected macrophages that contain different numbers (≤ 10, 10 – 20, ≥ 20) of MAP bacilli at 24 and 48 hours after infection (mean ± SEM), *n* = 13 (6 resistant and 7 susceptible).

Macrophages from resistant and susceptible animals had a similar infection rate after 24 and 48 h MAP infection as determined by auramine staining and fluorescent microscopy (Figure 5 and 6A). At 24 h, an average of 48.6% of macrophages from resistant animals and 50.5% of macrophages from susceptible animals were infected. At 48 h, the infection rate increased slightly for both resistant and susceptible macrophage cultures at 59.2% and 56.2% infected respectively.

Of the resistant and susceptible macrophages that became infected, the majority (mean > 80%) had phagocytosed less than 10 MAP organisms at 24 h after in vitro infection (Figure 6B). However, at 24 hpi, an average of 14% of infected macrophages from resistant animals contained 10 – 20 MAP bacilli compared to 7% of infected macrophages from susceptible animals but this difference was not statistically significant. The proportion of infected macrophages which had >20 MAP bacilli per cell was low for both types of animals at approximately 2%. At 48 hpi, the majority of infected macrophages from animals with either genotype still contained <10 MAP bacilli (Figure 6B). However, there was an increase in the number of infected macrophages from susceptible animals which had between 10 and 20 MAP per cell from an average of 7% to 19% of infected macrophages but this was not statistically significant. The equivalent condition in the macrophages from resistant animals remained relatively unchanged, with 14% of cells containing 10 to 20 MAP at 24 h and 13% of cells after 48 h infection. While the proportion of macrophages from resistant animals that contained >20 MAP also remained unchanged after 48 h at less than 3%, the number of infected macrophages from susceptible animals with >20 MAP per cell increased from a mean of less than 2% to more than 7% but this change did not reach statistical significance.

**Figure 7:**
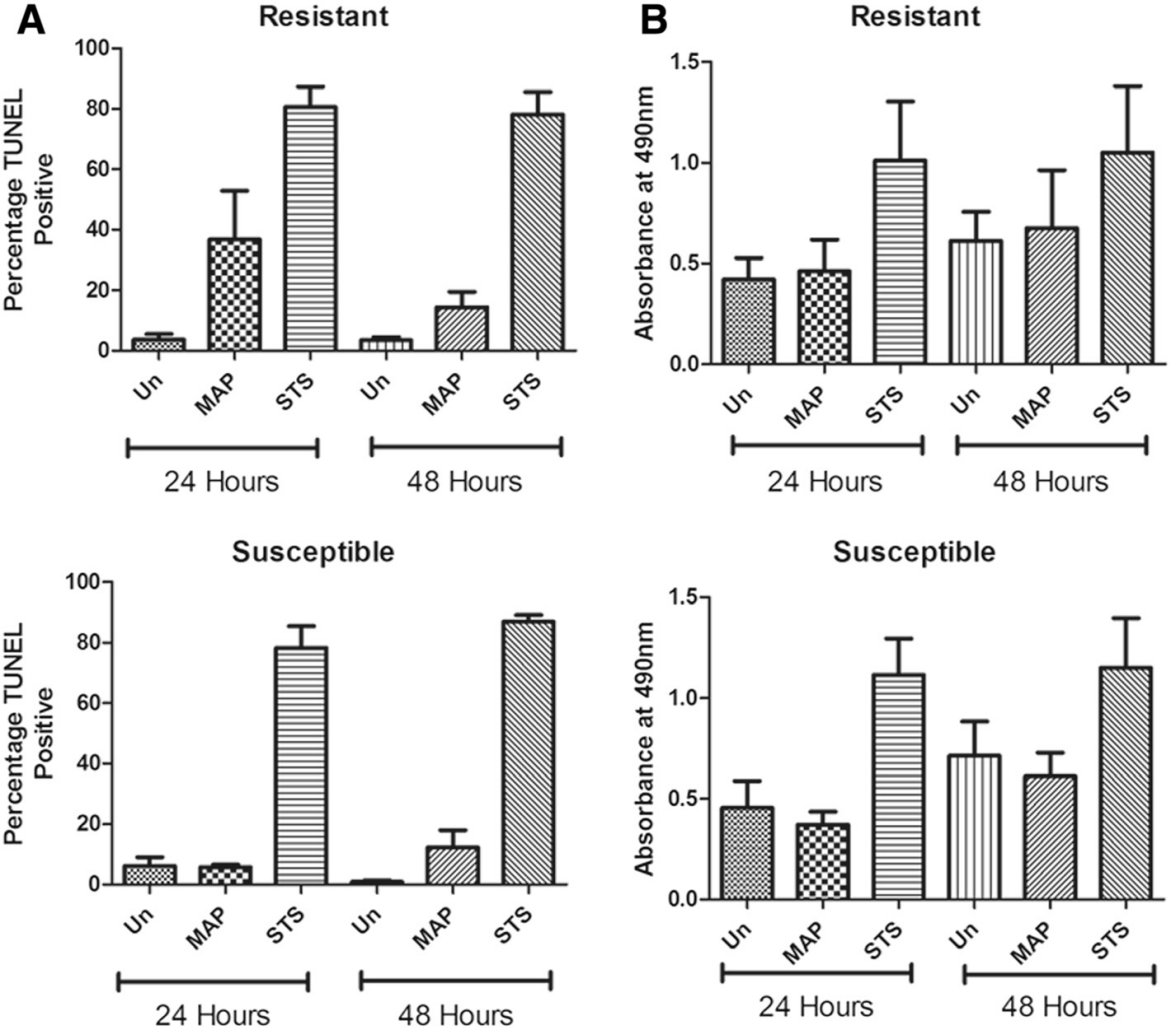
**Cell death in MDM from resistant or susceptible deer following in vitro MAP infection. **(**A**) Detection of apoptosis by TUNEL TMR red staining of MDM from crossbred animals of resistant or susceptible genotype at 24 or 48 h after MAP infection. Results are shown as mean percentage TUNEL positive cells ± SEM, *n* = 8 (4 resistant and 4 susceptible). Un = untreated, MAP = MAP-infected, STS = staurosporine at 200 ng/mL for 6 h (positive control). (**B**) Detection of total cell death by LDH release from MDM isolated from crossbred animals of resistant or susceptible genotype. Following 24 or 48 h MAP infection, supernatants of cultures were collected and analysed for LDH release. Results are given as mean absorbance values ± SEM, *n* = 13 (6 resistant and 7 susceptible).

Apoptotic events were detected by TUNEL staining coupled with fluorescent microscopy and the results of the various treatments on macrophages from resistant or susceptible animals are presented in Figure 7A. There was a very low level of apoptosis in untreated macrophages samples from both types of animals and at both time points, but macrophages from both animals showed the capacity for apoptosis induced by staurosporine treatment. Infection with MAP caused a greater proportion of macrophages from resistant animals to become apoptotic at 24 h than macrophages from susceptible animals; a mean of 37% of cells from resistant animals compared to a mean of 6% of cells from susceptible animals. However, this difference was not statistically significant and was observed at 48 h, as the rate of apoptosis in macrophages from animals of both genotypes was approximately 13%. No differences in total cell loss between slides from resistant animals and slides from susceptible animals were observed following MAP infection. The level of cell death of MAP-infected macrophages from resistant or susceptible animals, measured by LDH release, showed no statistically significant differences between the groups at either 24 or 48 hpi (Figure 7B).

## Discussion

Historically, infectious disease has played a major role in shaping the genetic make-up of populations due to natural selection [3]. Susceptibility and resistance to infectious disease shows marked variation in genetically diverse populations with some individuals expressing extremes of these phenotypes [36]. A goal of livestock breeding programs is to enhance the resistance of animals to infectious disease by selecting for resistant traits and excluding susceptible animals from these programs. The current study focused on Johne’s disease as a model to investigate the underlying biology of this resistance and susceptibility spectrum by targeting red deer which had a genetic bias towards either of these two poles.

In vitro infection of monocyte-derived macrophages with MAP resulted in quantitatively different gene expression profiles in animals with a resistant or susceptible genotype. Macrophages from genetically susceptible animals increased the expression of candidate inflammatory markers (iNOS, IL-1α, and IL-23p19, Figures 3 and 4), to a greater extent than the macrophages from genetically resistant animals. Other researchers have observed increased pro-inflammatory cytokine expression, particularly IFN-γ, IL-1α, TNF-α and IL-17 in the tissues of clinically diseased animals [22,37,38]. This suggests that the excessive transcription of inflammatory molecules by genetically susceptible animals observed here may lead to a dysfunctional or dysregulated innate immune response that is incompatible with the development of a protective adaptive immune response. In contrast, resistant animals may produce a more finely regulated and controlled increase in the expression of inflammatory markers in response to MAP which may be a precursor for the initiation of an appropriately controlled, protective adaptive immune response.

Given that immune responses to MAP in macrophages occur early and mycobacterial pathogens are known to express different antigens at different times in the infection process [39-41], the differences observed in gene expression between resistant and susceptible animals could also be a consequence of the sampling endpoint for the assays. At 24 h after infection, the macrophages from resistant animals may have dampened down the response which, initially, may have been considerable. By contrast, the macrophages from susceptible animals may have a persistently high inflammatory profile which could contribute to the activated state of these cells at 24 hpi. A dysregulated inflammatory response may fail to control the primary infection and trigger the immunopathology associated with chronic MAP infection. Monitoring immune markers at earlier and later time points than 24 h will be required to investigate this speculative hypothesis.

Macrophages from three animals of the purebred group displayed a very similar gene expression response to MAP to each other and to the macrophages of the resistant group. It is striking that these animals cluster so closely together and share the same sire which possessed a JBV close to neutral (0.04). On this basis, these animals were removed from the susceptible group and placed in a separate “intermediate” category (Figure 3). These animals were classified as susceptible due to the JBVs of their dams (> 0.3) but their macrophage gene expression responses suggest a strong genetic influence from their sire. While it would be interesting to explore the phenotype of these three “intermediate” animals, a limitation of the experiments presented here is that the study animals could not be classified as phenotypically susceptible or resistant. The functional phenotype could only be disclosed by experimental challenge or monitoring animals exposed to MAP infection naturally under field conditions, which was outside the scope of these experiments.

The expression of IL-10 remained relatively unchanged in response to MAP infection after 24 h and did not differ between the resistant and susceptible animals. IL-10 has been shown to be expressed at a higher level in clinically diseased animals compared to those that are sub-clinical or uninfected [42] as well as in bovine MDM isolated from infected cattle, in response to MAP challenge in vitro [17]. This cytokine is also reported to be expressed transiently in MDM from cattle and sheep early in the infection process [43,44]. The data reported here is limited to a single time point of 24 hpi so changes in IL-10 mRNA levels occurring earlier after infection would not have been detected. An alternative source of IL-10 following MAP infection in vivo may also be regulatory T cells as has previously been reported [45]. Another cytokine that may have benefited from earlier or later time points of analysis was IL-12p35 as the expression of this cytokine, while increasing in response to MAP, did not differentiate between resistant and susceptible animals. While IL-12p35 is a key cytokine promoting the Th1 pathway thought to be protective in mycobacterial diseases [23], the Th1 pathway has also been implicated in the pathology of Johne’s disease [22,46,47]. It is difficult to pinpoint the role of this Th1-associated cytokine in protection and disease with no separation in terms of gene expression of macrophages from resistant and susceptible animals.

Two groups of animals were used in this study to assess gene expression of macrophages from genetically resistant and susceptible red deer. The purebred animals showed statistically significant differences in gene expression between the resistant and susceptible genotypes. However, while the crossbred animals displayed similar trends to the purebred group, statistically significant differences were lacking between the resistant and susceptible genotypes. The greater scatter seen in the data obtained from the crossbred animals is compatible with the concept that disease resistance or susceptibility is due to small effects involving multiple genes rather than a single contributing genetic factor. Further, the lack of separation between the resistant and susceptible crossbred animals may be a result of the increased genetic heterogeneity of this group, the smaller groups size (*n* = 13 compared to *n* = 20 in the purebred group) or could be influenced by the different method used to categorise the animals as resistant or susceptible.

The use of an exotic model species such as red deer is challenging as protein detection reagents are either not available or have limited cross-reactivity in this species. To investigate functional differences between animals of a resistant or susceptible genotype, fluorescent staining and microscopy techniques were used to assay MAP phagocytosis rates as well as cell death rates. The rate of MAP infection at an MOI of 10:1 was approximately 50% at 24 and 48 hpi, for both types of animals (Figure 6A). Other researchers have noted similar infection rates of approximately 40-70% macrophage infection [48-50]. The majority of infected macrophages from resistant and susceptible animals had ingested less than 10 bacilli (Figure 6B). However, while the numbers of MAP within infected macrophages from resistant animals did not change over the 48 h time point, MAP numbers within macrophages from susceptible animals increased between 24 and 48 h. This could represent the replication of MAP within macrophages from susceptible animals while macrophages from resistant animals are able to prevent MAP multiplication. Further investigation with larger sample numbers is warranted to determine if this trend is significant.

Following MAP infection of macrophages in vitro there are three possible outcomes for the host cell: necrosis, a form of death characterized by plasma membrane disruption, apoptosis, a form of death in which plasma membrane integrity is preserved, or survival of MAP-infected macrophages [51]. The experiments described here were designed to distinguish these three outcomes at 24 and 48 hpi. In general, while the addition of MAP induced greater levels of macrophage apoptosis compared to uninfected controls, the proportion of apoptotic cells was low relative to the positive control (Figure 7A). MAP has been shown to induce apoptosis in bovine monocytes [52,53] but the levels are generally low as has been noted by Berger et al. in ovine MDM [44]. Several researchers have found that avirulent mycobacterial strains induce higher levels of apoptosis than virulent strains [54-56]. Consequently, apoptosis has been proposed to be an innate defence mechanism which can be subverted by virulent mycobacteria [56,57]. However, macrophage apoptosis can be increased when a higher MOI of virulent mycobacteria is used in the infection protocol although it has been noted that the apoptotic process quickly converts to a necrotic cell death modality [58,59]. A greater proportion of macrophages from resistant animals were apoptotic following MAP infection for 24 h compared to the equivalent treatment in the macrophages from susceptible animals (Figure 7A) but this effect was not evident at 48 hpi. It is possible that the macrophages from a resistant animal use apoptosis as an innate defence mechanism in the early stages of infection by depriving MAP of its preferred growth niche, by broadening the activation of other immune cells such as dendritic cells through efferocytosis and by direct anti-mycobacterial activities found in apoptotic macrophages.

### Conclusions

Control of mycobacterial infection requires a properly balanced and regulated cytokine environment within infected tissues. The data obtained in this study infers that a dysregulated immune response characterised by excessive inflammatory gene expression occurs in macrophages from susceptible animals. This may pre-empt the development of protective immunity. In the absence of an appropriate adaptive immune response, MAP infection could invoke the chronic immunopathology observed in clinical Johne’s disease. In contrast, macrophages from resistant animals, while expressing the same inflammatory genes as those cells from susceptible animals, do so at a significantly lower level as well as exhibiting higher rates of apoptotic cell death. Together, this implies that controlled regulation of inflammation may be pivotal to protection against chronic inflammatory bowel disease caused by MAP infection.

## Competing interests

The authors declare that they have no competing interests.

## Authors’ contributions

BD conducted the experiment, analysed the data, and drafted the manuscript. SL contributed the data in Figure 1. RO and FG assisted in the experimental design and contributed to the interpretation of results. All authors read and approved the final manuscript.
